# Composite Coatings of AMg3 Alloy Formed by a Combination of Plasma Electrolytic Oxidation and Fluoropolymer Spraying

**DOI:** 10.3390/molecules28020465

**Published:** 2023-01-04

**Authors:** Dmitry V. Mashtalyar, Konstantine V. Nadaraia, Igor M. Imshenetskiy, Evgeniy A. Belov, Mariia S. Gerasimenko, Sergey L. Sinebryukhov, Sergey V. Gnedenkov

**Affiliations:** 1 Department of Electrochemical Systems and Processes of Surface Modification, Institute of Chemistry FEB RAS, 159 pr. 100-Letiya Vladivostoka, 690022 Vladivostok, Russia; 2 Institute of High Technologies and Advanced Materials, Far Eastern Federal University, 10 Ajax Bay, Russky Island, 690922 Vladivostok, Russia

**Keywords:** aluminum alloy, plasma electrolytic oxidation, composite coatings, corrosion, wear, hydrophobicity

## Abstract

This paper presents the results of an investigation of the changes in the corrosion, wear resistance, and wettability of composite coatings formed on the AMg3 alloy through plasma electrolytic oxidation (PEO) and subsequent spraying with an organofluorine polymer. The evaluation of the electrochemical properties of the composite layers revealed a decrease in the corrosion current density compared with the PEO coating (from 3.8 × 10^−8^ to 3.1 × 10^−11^ A/cm^2^). The analysis of the wear resistance of composite coatings established that the application of this type of coating reduced the wear of the samples by two orders of magnitude when compared with the PEO layer. Using the contact-angle measurement, it was found that with an increase in the number of polymer spray applications, the wettability of coatings decreased, so the contact angle for the composite coating with triple fluoropolymer application increased by 134.3° compared to the base PEO coating.

## 1. Introduction

Aluminum alloys remain the basic structural materials for various industries, such as aerospace, oil and gas, construction, and others [1,2,3,4] due to their low density, complex performance characteristics, high manufacturability, and weldability. Their composition, structure, and manufacturing methods, including thermomechanical processing, continue to be constantly improved in accordance with the increasing requirements for the structures using these alloys [5,6,7]. Among the various aluminum alloys, the composition of AMg3 alloy has the most optimal magnesium content, which provides excellent deformability in hot and cold conditions and a lower specific gravity value than pure aluminum. However, the natural oxide film on the surface of the material is not sufficiently protective in aggressive corrosive environments. The main method for protecting metals, including aluminum, is the formation of coatings on the surface of a part or product. One of the promising ways to form such protective coatings is plasma electrolytic oxidation (PEO) [8,9,10,11]. The coatings obtained using PEO have highly protective and wear-resistant properties, high adhesion to the metal substrate, as well as a developed surface with pores; this makes it possible to modify such coatings (by incorporating the various components into the pores) with different materials, depending on the purpose of future use [10,11,12]. The purpose of this work is to modify such coatings previously formed on AMg3 alloy by introducing superdispersed polytetrafluoroethylene (SPTFE) into their structure using a spray-coating method. This modification was carried out in order to improve the surface properties of the samples, increase their wear resistance, and also impart hydrophobic properties to the surface. To the best of the authors’ knowledge, the approach of spraying SPTFE onto the PEO coatings formed on aluminum alloys has never been used before. By changing the speed and time of spraying, and the flow rate and pressure of the compressor, this method forms composite layers directionally. Such coatings are able to increase the protective characteristics compared with the original PEO layers. The composite fluoropolymer-containing coatings obtained in the course of this study can be used in a number of industries. The corrosion resistance of the coatings may be required not only in the case of direct contact between the materials in an aggressive environment but also when it is used in regions with a humid marine climate. Given the insufficient wear resistance of aluminum and its alloys, the formed coatings can be used in any moving parts made of aluminum materials. In turn, this will help to increase the service life of such parts without loss of functional quality, and, consequently, reduce the economic costs associated with replacement, repair, and equipment downtime. Thus, the developed method for forming multifunctional surfaces can significantly increase the economic efficiency in aerospace, chemical, and automotive industries, as well as in shipbuilding and ship repair, etc.

## 2. Results and Discussion

Analysis of the literature data revealed a high level of protection provided by composite coatings formed based on PEO layers using polytetrafluoroethylene. In previous works, a method of forming polymer-containing layers was developed by dip-coating a sample in a suspension of superdispersed polytetrafluoroethylene [13]. Protective coatings significantly increase the resistance of alloys to corrosion in a NaCl solution. Composite coatings formed through the triple application of superdispersed polytetrafluoroethylene have unique anticorrosive properties, reducing the value of the corrosion current density for the protected alloy to 1.6 × 10^–11^ А/cm^2^, which is more than three orders of magnitude lower than that for PEO coatings and five orders of magnitude lower than that for the uncoated sample. However, in some cases, dip-coating for the creation of a composite polymer-containing layer is difficult to apply due to the large consumption of material and technological difficulties, and it is also impossible to achieve high hydrophobic properties of the surface using this method. This arises due to a more developed surface, which is achieved with the spraying method [14]. In this regard, we developed a method for the formation of polymer-containing layers in which the fluoropolymer component was applied to the base PEO coating by spraying [14].

### 2.1. Coatings Microstructure and Composition

**Figure 1:**
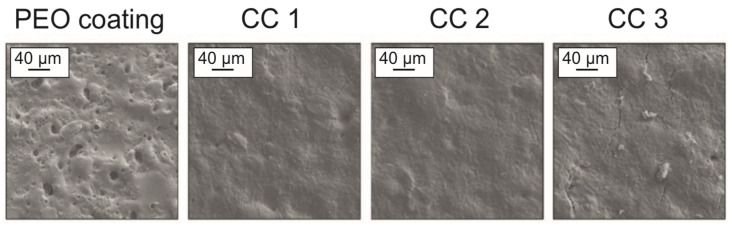
SEM images of the PEO and composite coatings on aluminum alloy.

Analysis of the SEM images of the PEO and composite coatings allowed us to establish that the complete sealing of the coating pores occurred with a small amount of the sprayed substance (Figure 1).

**Figure 2:**
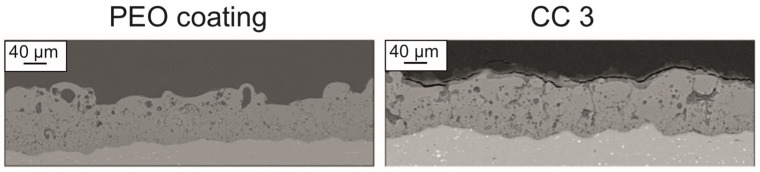
SEM images of cross-section of PEO coating and CC 3 on aluminum alloy.

The analysis of the SEM image of the cross-section of PEO coating and CC 3 indicated the incorporation of the polymer into the porous part of the PEO layer, which means that the formed coatings were composite. Additionally, the results of the analysis of the cross-section coatings in Figure 2 made it possible to reveal a decrease in the distribution of pores within the coatings *P_cs_* with an increase in the amount of the sprayed substance due to filling mainly the upper porous layer of the PEO coating. The coatings formed on aluminum had a small surface porosity (Figure 1), and due to the peculiarities of the PEO layer formation process on aluminum, a significant number of pores were distributed in the middle part of the coating (Figure 2). Additionally, a high *P_cs_* index resulted from the high coating thickness (Table 1).

**Figure 3:**
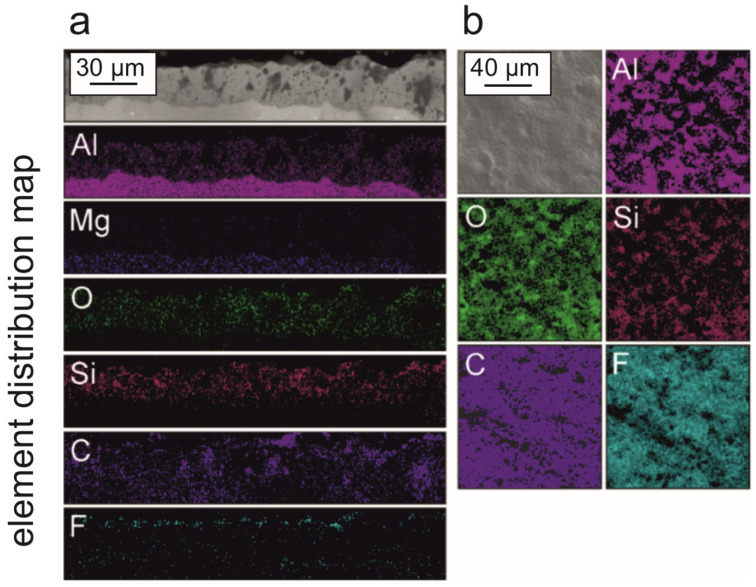
Results of energy-dispersion spectroscopy of composite coatings on aluminum alloy: within the cross-section (**a**) and over the surface (**b**).

The results of energy-dispersion spectroscopy (EDS) (Figure 3) allowed us to establish the distribution of elements within the coating and on its surface. Thus, in the samples, the presence of aluminum, oxygen, and silicon in the composition of the PEO coating was observed, distributed within the thickness and over the surface of the coating, while fluorine and carbon were present only in the outer part, which confirmed the presence of organofluorine compounds in the composition of the composite coating (Figure 3). The presence of magnesium on the element distribution map is explained by the composition of the AMg3 alloy (up to 3.8 wt.% Mg content).

**Figure 4:**
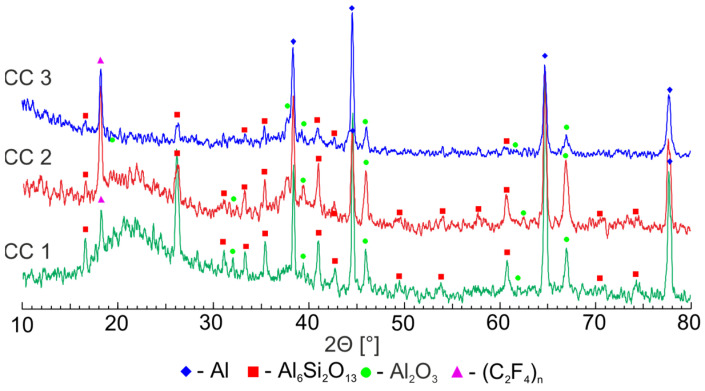
XRD patterns of composite coatings formed on aluminum alloy.

The phase composition of the formed coatings was determined using X-ray diffraction (Figure 4). For composite coatings, general trends were observed, namely an increase in the intensity of the polytetrafluoroethylene and a decrease in the intensity of the phase, observed in the PEO coating (Al_2_O_3_ and Al_6_Si_2_O_13_) as the number of spraying applications increased (Figure 4).

Consequently, summarizing the data of XRD and EDS analyses, it can be concluded that based on the PEO coating consisting mainly of phases and oxide (Al_2_O_3_) and metal and electrolyte compounds, as a result of treatment with organofluorine dispersions by means of spraying, composite coatings with a high content of crystalline polytetrafluoroethylene (Figure 4), incorporated in the outer porous layer of the PEO coating (Figure 3), were formed.

### 2.2. Electrochemical Properties

**Figure 5:**
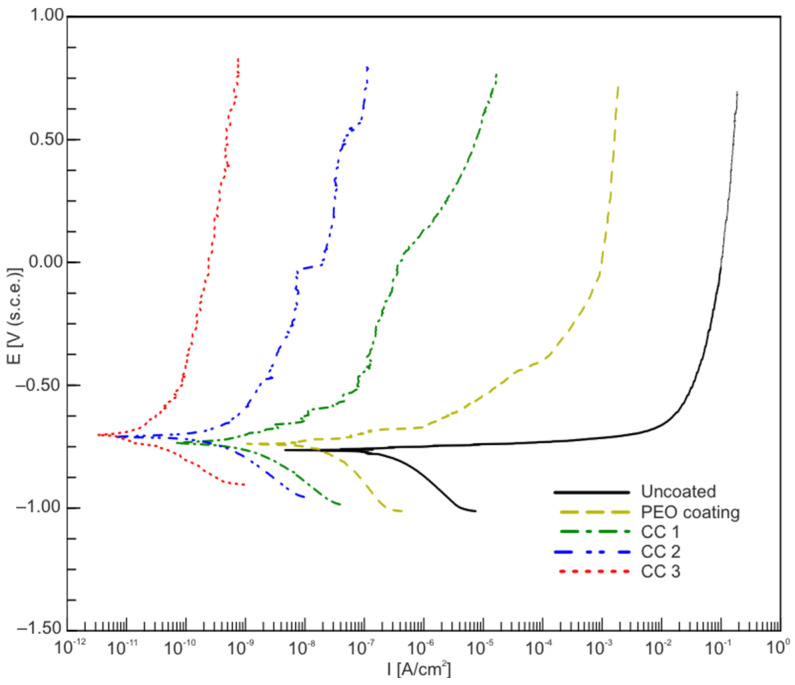
Polarization curves for samples made of AMg3 aluminum alloy with various types of surface treatment.

The corrosion properties of the samples were investigated using the potentiodynamic polarization technique (Figure 5). The analysis of the results of electrochemical studies revealed that the sample with PEO coating significantly reduced the corrosion current density *I_C_* and increased the polarization resistance *R*_P_ (Figure 5, Table 2), compared with the alloy without coating. According to the data presented, a single application of a fluoropolymer (CC 1) reduced the corrosion current density by one order of magnitude in comparison with a PEO coating (Table 2). An increase in the frequency of processing with SPTFE (CC 2) led to a decrease in the corrosion current density by more than four times in comparison with CC 1 (Table 2). The highest protective properties were demonstrated by the samples using a threefold spraying treatment of a fluoropolymer suspension and subsequent heat treatment. Compared with the uncoated and PEO-coated samples, the corrosion current density for CC 3 decreased by four and three orders of magnitude, respectively (Table 2).

**Figure 6:**
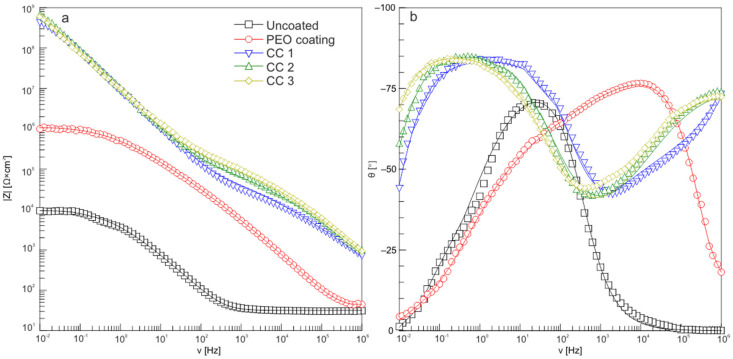
Bode plots (dependence of impedance modulus |Z| (**a**) and phase angle *θ* (**b**) on frequency) for samples made of AMg3 aluminum alloy with various types of surface treatment.

A comparative analysis of the Bode plot for the samples made of aluminum alloy with various types of surface treatment (Figure 6) allowed us to evaluate the changes in the protective characteristics of the obtained coatings. For the uncoated sample, a characteristic minimum was observed in the region of medium frequencies. The formation of a protective PEO coating on the surface led to the appearance of a second band in the high-frequency region, which characterized the resistance of the porous PEO layer. The incorporation of a fluoropolymer into the composition of PEO coating led to a significant change in the shape of the dependence, namely an increase in the impedance modulus at low frequencies.

**Figure 7:**
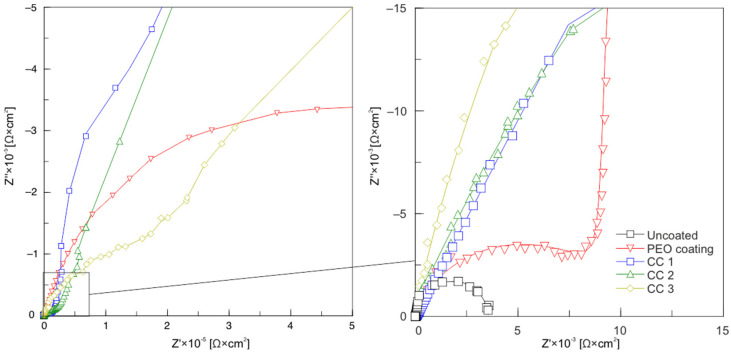
Nyquist plots for uncoated, PEO-coated, CC 1, CC 2, and CC 3 samples.

Electrochemical impedance spectroscopy studies for samples with various types of surface treatment are presented as Nyquist plots in Figure 7. Based on the analysis of these plots, it can be concluded that the loop diameter of the high-frequency part in the plots revealing the dependence of the imaginary part of the impedance, *Z″*, on the real part of the impedance, *Z′*, for the uncoated sample was much smaller than the coated samples, which confirms the results of potentiodynamic tests (Figure 7, Table 2). The plot for a sample with a PEO coating showed a smaller loop than samples with composite layers; consequently, it was less protective.

### 2.3. Wear Resistance

A single application of a fluoropolymer material to the PEO layer did not significantly affect the wear resistance of the resulting coating CC 1 (Table 3). This was due to the structural features of PEO coatings on aluminum; most of the polymer penetrated deep into the pores during heat treatment, as a result of which the surface of CC 1 was not covered with a continuous polymer film. An increase in the frequency of fluoropolymer treatment significantly increased its wearproof ability by one and three orders of magnitude, respectively, for samples with a double and triple application of the polymer in comparison with samples with PEO coating and CC 1 (Table 3).

The analysis of the data obtained during the scratch testing of the PEO layer formed on the AMg3 aluminum alloy indicated high values of adhesion of the protective coating to the substrate (Table 4). For PEO coatings, the load value at which the beginning of peeling of the coating was observed *L*_C2_ was 13.0 N.

The application of organofluorine materials by means of spraying led to an insignificant increase in the *L*_C2_ value (Table 4). An increase in the amount of introduced fluoropolymer also affected the amount of load at which the abrasion of the coating to the substrate, *L*_C3_, occurred, increasing it to 17.1 N (Table 4). The observed effect is explained by the fact that SPTFE fills the pores and defects of the PEO layer, providing the composite coating with a more uniform distribution of the load when it is scratched and, in general, increasing the adhesive strength of the coating by reducing the internal stresses arising under the influence of the load.

### 2.4. Microhardness of Composite Coatings

**Figure 8:**
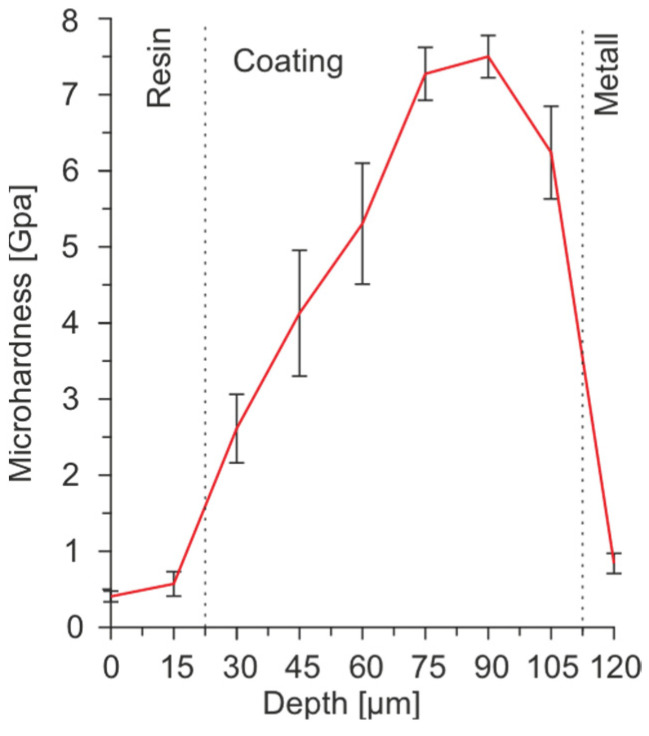
Distribution of microhardness over the thickness of PEO coating on AMg3 aluminum alloy.

The thickness of the polymeric layer is 1–2 microns, depending on the amount of embedded PTFE and the substrate material [15]. Due to the softness and insignificant thickness of the polymeric film, the microhardness of composite and PEO coatings is indistinguishable [16]. In this regard, the elastoplastic properties of the PEO layers were studied in this paper. Figure 8 shows the plots of changes in microhardness over the thickness of PEO coatings on the AMg3 alloy.

The analysis of the distribution of microhardness over the thickness of coatings revealed the positive effect of PEO coating on the value of microhardness. An increase in this parameter was observed deep into the coating. In the nominal center of the PEO layer, the microhardness values reached a maximum, after which a decrease in this parameter was observed as moving toward the PEO coating/metal boundary. The hardness of the protective coating increased with a thickness of 22 µm (Figure 8) from the metal boundary. In this area, the coating was the most homogeneous and contained fewer pores and defects that could lead to the destruction of the layer.

### 2.5. Wettability

The treatment of PEO coating on the AMg3 aluminum alloy with a fluoropolymer allowed for the study of hydrophobic properties by comparing its values with those of the resulting composite coatings. Moreover, it can be concluded that for these coatings, with an increase in the frequency of treatment with a fluoropolymer material, the water wettability decreased. CC 1 had a CA significantly less than CC 2 and CC 3 (Table 5), which can be explained by the structure and composition of these coatings.

## 3. Materials and Methods

Alloy plates of AMg3, with a size of 20 × 30 × 1.5 mm^3^, were used as samples. The specimens were ground with SiC papers, with a reduction in the grain size of the abrasive to 15 μm, and were additionally polished with aluminum oxide paper with a grain size of 3 μm. After polishing, the samples were washed with deionized water, degreased with alcohol using an ultrasonic bath RK31 (Bandelin Electronic, Berlin, Germany), and air-dried. Then, the samples were coated with the PEO method using a bipolar potentiodynamic mode (Table 6) and electrolytic systems of complex composition (NaF + Na_2_B_4_O_7_ × 10H_2_O + C_4_H_4_O_6_K_2_·0.5H_2_O + Na_2_SiO_3_·5H_2_O) [17,18]. To create composite layers as the polymer component in this work, superdispersed polytetrafluoroethylene of the Forum^®^ trademark (Institute of Chemistry FEB RAS, Vladivostok, Russia) was used, obtained by A.K. Tsvetnikov and A.A. Uminsky using the method of the thermogradient synthesis of F4 fluoroplast. In order to increase the manufacturability of the composite layer application, a 15% suspension of SPTFE powder in isopropyl alcohol was used in this work [13,19]. The polymer was sprayed, and subsequently, the sample was heat-treated at 350 °C for 15 min. We studied the influence of the number of polymer deposits on the properties of the coatings, depositing the polymer one, two, and three times on the base PEO layer. These samples are further designated in the text as CC 1, CC 2, and CC 3, respectively.

For surface morphology, a Sigma (Carl Zeiss, Jena, Germany) scanning electron microscope (SEM) was used. To evaluate the porosity of the coatings, the SEM images were processed using the ImageJ software (National Institutes of Health, Rockville Pike, Bethesda, MD, USA). The porosity *P* of the coatings was calculated as the percentage of the area occupied by the pores to the total area. For processing the SEM images with ImageJ, the sensitivity threshold was chosen so that all the pores in the coating were visibly marked.

For the qualitative assessment of the presence of organofluorine polymer in the composition of coatings, the samples were examined using X-ray diffraction (XRD). X-ray diffraction was performed using an automatic X-ray diffractometer D8 Advance (Bruker, Karlsruhe, Germany) with CuK_α_ radiation. The Bragg–Brentano geometry focusing was used in the range of 2*θ* angles from 10° to 80°, with a scanning step of 0.02° and an exposure time of 1 s at each point. For the analysis of the obtained XRD patterns, the search program “EVA” with the data bank “PDF-2” was used.

The electrochemical properties of the samples were studied using the VersaSTAT MC electrochemical system (Princeton Applied Research, Princeton, NJ, USA). The measurements were carried out in a three-electrode cell at room temperature in a 3.5% NaCl solution. The platinized niobium mesh was used as a counter electrode. The saturated calomel electrode (SCE) was used as a reference electrode. The exposed surface area of the samples was 1 cm^2^. Before the start of electrochemical measurements, in order to achieve steady-state conditions, the samples were kept in the solution for 900 s [20,21]. For impedance measurements, a sinusoidal signal with 10 mV (rms) amplitude was used. The spectra were recorded from 1 MHz to 0.01 Hz with a scan rate of 7 points/decade. A potentiodynamic polarization test was performed at a sweep rate of 1 mV/s. The potential was changed in the range from *E_C_* − 0.25 V to *E_C_* + 1.5 V. The Levenberg–Marquardt approach [22] was used to calculate the corrosion parameters of the studied samples by fitting the experimental data (i.e., current density *I* vs. potential *E*) using Equation (1):

(1) $$I=I_{C}\left(10^{\frac{E-E_{C}}{\beta_{a}}}+10^{-\frac{E-E_{C}}{\beta_{c}}}\right).$$

This method makes it possible to obtain the best fit values of corrosion potential, *E_C_*, corrosion current density, *I_C_*, and the cathodic and anodic Tafel slopes, *β_c_* and *β_a_*.

The polarization resistance, *R_P_*_,_ was determined in a separate experiment using a linear polarization resistance test via potentiodynamic polarization at a sweep rate of 0.167 mV/s in the potential region Δ*E = Е_C_* ± 20 mV, in which the linear dependence *I = f(E)* was observed.

Calculation of *R_P_* values was carried out according to Equation (2):

(2) $$R_{P}=\frac{\Delta E}{\Delta I}.$$

The wearproof ability of the formed coatings was investigated using a TRB-S-DE Tribometer (CSM Instruments, Peseux, Switzerland). The test was carried out at room temperature in a dry friction mode at a sliding speed of 50 mm/s and a load of 10 N. A corundum ball (α-Al_2_O_3_) was used as a counterbody. The tests were continued until the corundum ball reached the metal. The profile of the coating wear track was measured using a Surtronic 25 Profilometer (Taylor Hobson Ltd., Leicester, UK). The wear rate was calculated using Equation (3):

(3) $$P=\frac{\Delta V_{sample}}{NF},$$

where *P* is the value of the wear rate ((m^3^ 10^−9^)/(N m)), Δ*V* is the volume loss of the sample during testing (m^3^ 10^−9^), *N* is the wear track length (m), and *F* is the applied load (N).

The volume loss of the samples was calculated according to Equation (4):

(4) ΔV=SL,

where *L* is the circumference of the abrasion track (m), and *S* is the cross-sectional area of the wear track (m^2^ 10^−6^).

The adhesive characteristics of the coatings were evaluated using a Revetest Scratch Tester (CSM Instruments, Peseux, Switzerland). The study of adhesion via scratching was carried out by measuring the critical load at which the destruction of the coating was observed. The indenter was a conical diamond tip (Rockwell type) with an angle at the top of 120° and a radius of 200 microns. The path of the movement of the indenter along the surface of the sample was 5 mm, and the maximum applied load was 20 N.

The microhardness of the coatings was measured using a dynamic ultramicrohardometer DUH–W201 (Shimadzu, Kyoto, Japan). The universal microhardness *H_µ_* was measured on the transverse section of the sample using a Vickers indenter at a load of 100 mN.

The wettability of the studied materials was evaluated with the sessile drop method [23] on a DSA100 device (KRÜSS, Hamburg, Germany). During the test, the contact angle (CA—*θ*) was measured as the angle between the baseline and tangent to the droplet’s outline at a three-phase point [24,25]. To calculate the CA, the Young–Laplace method was used, considering the gravitational distortion of the liquid droplet formed under its own weight [25].

The contact angle hysteresis (CAH—*θ_CAH_*) was calculated in accordance with Equation (5) [25], where *θ_a_* and *θ_r_* are the advancing and receding contact angles, respectively, measured in accordance with the procedure described in [26]. The volume of the initial drop was equal to 10 µL. Deionized water was gradually dosed into the drop (at a rate of 0.05 µL/s). The angle *θ_a_* was measured when the shape of the drop did not change, and the contact line began to increase. After the measurement of *θ_a_*, aspiration was performed at a rate of 0.05 µL/s. The measurement of *θ_r_* was performed when the shape of the droplet did not change during aspiration, and the contact line decreased.

(5) $$\theta_{CAH}=\theta_{a}-\theta_{r}.$$

## 4. Conclusions

According to the results of the conducted studies, it can be concluded that SPTFE spraying on the PEO-coated AMg3 aluminum alloy significantly improved the protective properties of the treated material. The presence of crystalline polytetrafluoroethylene in the composite coating composition was confirmed by XRD studies. The decrease in the corrosion current density for samples with the composite coatings obtained as a result of a triple spraying application of the superdispersed polytetrafluoroethylene suspension was more than four and three orders of magnitude higher than samples without coating and those with PEO layers, respectively. The presence of the polymer with a low coefficient of friction in the composition of the formed coating improved the wearproof capability of the material by two orders of magnitude, compared with the PEO layer. In addition, the incorporation of the fluoropolymer into the composition of the base PEO layer allowed a hydrophilic surface to exert hydrophobic properties.

## Figures and Tables

**Table 1:** Thickness and porosity of PEO and composite coatings formed on aluminum alloy (*d* is a coating thickness, and *P_sur_* and *P_cs_* are surface porosity and porosity within the coatings’ cross-section, respectively).

Sample	*d* [µm]	*P_sur_* [%]	*P_cs_* [%]
PEO coating	110 ± 3	5.12	17.41
СС 1	112 ± 2	1.13	17.28
СС 2	112 ± 1	-	17.04
СС 3	114 ± 2	-	16.28

**Table 2:** Corrosion properties (corrosion potential, *E_C_*, corrosion current density, *I_C_*, polarization resistance, and *R*_P_) of samples made of AMg3 aluminum alloy with various types of surface treatment.

Sample	*E_C_* [V]	*I_C_* [А/cm^2^]	*R_P_* [Ω cm^2^]
Uncoated	−0.75	6.1 × 10^−7^	2.9 × 10^4^
PEO coating	−0.74	3.8 × 10^−8^	9.1 × 10^5^
СС 1	−0.73	1.1 × 10^−9^	2.8 × 10^7^
СС 2	−0.71	4.4 × 10^−10^	1.2 × 10^8^
СС 3	−0.71	3.1 × 10^−11^	1.8 × 10^9^

**Table 3:** The wear of samples made of AMg3 aluminum alloy with various types of surface treatment.

Sample	Number of Cycles	Wear [(m^3^ 10^−9^)/(N·m)
PEO coating	84	3.1 × 10^−2^
СС 1	257	1.7 × 10^−2^
СС 2	5,168	1.1 × 10^−3^
СС 3	43,529	9.4 × 10^−5^

**Table 4:** Critical loads for coatings obtained on AMg3 aluminum alloy.

Sample	*L*_C2_ [N]	*L*_C3_ [N]
PEO coating	13.0 ± 0.2	15.5 ± 0.4
СС 1	13.2 ± 0.3	16.2 ± 0.4
СС 2	13.9 ± 0.2	17.1 ± 0.5
СС 3	13.9 ± 0.2	17.1 ± 0.5

**Table 5:** Wettability of the AMg3 aluminum alloy samples with various types of surface treatment: *θ*—contact angle, *θ_CAH_*–contact angle hysteresis, *θ_a_*–advancing contact angle, and *θ_r_*–receding contact angle.

Sample	*θ* (°)	*θ_a_* (°)	*θ_r_* (°)	*θ_CAH_* (°)
Uncoating	64.8 ± 1.8	–	–	–
PEO coating	7.9 ± 2.4	–	–	–
СС 1	124.7 ± 2.6	121.5 ± 0.2	94.8 ± 0.3	26.7 ± 0.5
СС 2	138.4 ± 2.9	152.6 ± 0.3	116.5 ± 0.9	36.1 ± 1.2
СС 3	142.2 ± 2.7	151.2 ± 0.2	110.5 ± 0.3	40.7 ± 0.5

**Table 6:** Plasma electrolytic oxidation mode for AMg3 aluminum alloy (t—process time).

Process Stage	I	II	III
	(t *=* 300 s)	(t *=* 2000 s)	(t *=* 400 s)
Anode phase (V)	from 30 to 450	from 450 to 480	from 480 to 400
Cathode phase (A)	from –1 to –5	from –5 to –10	from –10 to –1

## Data Availability

Data are available upon request.
